# Histidine-rich glycoprotein as a novel predictive biomarker of postoperative complications in intensive care unit patients: a prospective observational study

**DOI:** 10.1186/s12871-022-01774-7

**Published:** 2022-07-20

**Authors:** Masahiko Oiwa, Kosuke Kuroda, Naoya Kawanoue, Hiroshi Morimatsu

**Affiliations:** Department of Anesthesiology and Resuscitology, Okayama University Graduate School of Medicine, Dentistry and Pharmaceutical Sciences, 2-5-1, Shikata-cho, Kita-ku, Okayama, 700-8558 Japan

**Keywords:** Biomarker, Clavien–Dindo classification, Histidine-rich glycoprotein, Intensive care unit, Perioperative management, Postoperative complication, Predictor

## Abstract

**Background:**

Decrease in histidine-rich glycoprotein (HRG) was reported as a cause of dysregulation of the coagulation-fibrinolysis and immune systems, leading to multi-organ failure, and it may be a biomarker for sepsis, ventilator-associated pneumonia, preeclampsia, and coronavirus disease 2019. However, the usefulness of HRG in perioperative management remains unclear. This study aimed to assess the usefulness of HRG as a biomarker for predicting postoperative complications.

**Methods:**

This was a single-center, prospective, observational study of 150 adult patients who were admitted to the intensive care unit after surgery. Postoperative complications were defined as those having a grade II or higher in the Clavien–Dindo classification, occurring within 7 days after surgery. The primary outcome was HRG levels in the patients with and without postoperative complications. The secondary outcome was the ability of HRG, white blood cell, C-reactive protein, procalcitonin, and presepsin to predict postoperative complications. Data are presented as number and median (interquartile range).

**Results:**

The incidence of postoperative complications was 40%. The HRG levels on postoperative day 1 were significantly lower in patients who developed postoperative complications (*n* = 60; 21.50 [18.12–25.74] µg/mL) than in those who did not develop postoperative complications (*n* = 90; 25.46 [21.05–31.63] µg/mL). The Harrell C-index scores for postoperative complications were HRG, 0.65; white blood cell, 0.50; C-reactive protein, 0.59; procalcitonin, 0.73; and presepsin, 0.73. HRG was independent predictor of postoperative complications when adjusted for age, the presence of preoperative cardiovascular comorbidities, American Society of Anesthesiologists Physical Status Classification, operative time, and the volume of intraoperative bleeding (adjusted hazard ratio = 0.94; 95% confidence interval, 0.90–0.99).

**Conclusions:**

The HRG levels on postoperative day 1 could predict postoperative complications. Hence, HRG may be a useful biomarker for predicting postoperative complications.

**Supplementary Information:**

The online version contains supplementary material available at 10.1186/s12871-022-01774-7.

## Background

After surgery, approximately 3–22% of patients develop postoperative complications [1–4]. Once a complication develops, the patient’s hospitalization is prolonged, worsening the prognosis [1]. Previous studies have reported that white blood cell (WBC), C-reactive protein (CRP), procalcitonin (PCT), and presepsin (P-SEP) levels may be useful biomarkers for predicting postoperative complications [5–13]. However, standard biomarkers for predicting postoperative complications have not been established.

Histidine-rich glycoprotein (HRG) is an approximately 75-kDa glycoprotein mainly synthesized in the liver and present at a level of 60–150 µg/mL in healthy individuals [14–17]. HRG binds to various ligands and regulates coagulation fibrinolysis, the immune system, and angiogenesis [17, 18]. In mice with sepsis, HRG levels decreased because of decreased production in the liver and increased degradation. Furthermore, decrease in HRG levels caused dysregulation of the coagulation-fibrinolysis system, abnormal neutrophil morphology, endothelial cell abnormalities, and immune thrombosis, leading to multiple organ failure [17, 19]. Earlier clinical studies have reported that decrease in HRG levels may be a biomarker for sepsis [16, 20], ventilator-associated pneumonia [21], preeclampsia [22], and coronavirus disease 2019 [23].

However, the usefulness of HRG in perioperative management remains unknown. We hypothesized that HRG levels on postoperative day 1 (POD 1) could predict postoperative complications and conducted a prospective observational study to assess the usefulness of HRG as a biomarker for predicting postoperative complications.

## Methods

### Study design and ethical considerations

This single-center, prospective, observational study was approved by the Institutional Review Board of the Okayama University Hospital (Okayama, Japan) on August 14, 2020 (approval number: 2007–006). The need for registration of the study was waived because this was an observational investigation. The requirement for written informed consent was waived by the Institutional Review Board because this was a non-invasive study using residual blood samples collected from routine blood tests performed on POD 1. We described the study protocol to the all patients and obtained verbal informed consent for study participation and publication were obtained from them. This information was preserved as an electronic medical record before their inclusion in the study. The patients received a copy of the study description and were provided with contact information, in case additional questions or concerns arose. In addition, the study protocol was published on the website. We followed the Strengthening the Reporting of Observational Studies in Epidemiology guidelines [24].

### Patients and data collection

Patients admitted to the intensive care unit (ICU) after surgery at Okayama University Hospital (Okayama, Japan) during consecutive periods were prospectively included. At our institution, all patients post respiratory surgery, neurosurgery, hepato–biliary–pancreatic surgery, esophageal surgery, cardiovascular surgery, and highly invasive oral and otolaryngological surgery are admitted to the intensive care unit. In other departments, patients are admitted to the intensive care unit post-surgery at the discretion of the physician. According to previous studies, HRG levels are higher in adults than in children [15] and decrease during pregnancy [22]; thus, patients who were pregnant or < 20 years old were excluded. We planned to enroll 150 patients based on a power calculation. According to our previous study [16], we expected that the HRG levels would vary by 20 µg/mL between patients with and without postoperative complications; this calculation was based on the number of patients required for an 80% power to detect a 20 µg/mL difference in HRG levels. A two-sided type I error of 0.05 was considered for the 10% incidence of postoperative complications and loss to follow up.

All enrolled patients’ information was collected from electronic medical records. Preoperative comorbid cardiovascular diseases included arrhythmia, coronary artery disease, heart failure, and macrovascular diseases. Chronic kidney disease was classified with an estimated glomerular filtration rate < 50 mL/min. The surgical Apgar score (SAS) was calculated using anesthesia records. Preoperative and postoperative sequential organ failure assessment scores and acute physiology and chronic evaluation II scores on admission to the ICU were calculated using clinical variables and blood-test results.

Postoperative complications were defined as an extended Clavien–Dindo classification [25] grade II or higher, occurring within 7 days after surgery. Among the postoperative complications, we defined infectious complications as those that required antibiotic therapy or drainage due to infection. The mortality rate was assessed 28 days postoperatively. The enrolled patients were followed up to the day of discharge or 28 days postoperatively.

### Measurement methods

To measure HRG levels, we used the residual blood samples collected for routine blood tests in tubes containing K2-EDTA in the morning of POD 1. The samples were then centrifuged at 3,000 rpm for 10 min. Plasma components were transferred to polypropylene tubes with a pipette, and a protease inhibitor cocktail (Complete mini EDTA-free; Roche Diagnostics, Basel, Switzerland) was added. The samples were stored at -80 °C.

Plasma HRG levels were measured using a modified quantitative sandwich enzyme-linked immunosorbent assay, in which the detection and chromogenic reagents were changed from those previously described [16] because of discontinuation of the reagent. In brief, a rat monoclonal antibody (mAb) against human HRG (made in-house, number 75–14) was used as the capture antibody, and a nickel (Ni ^2+^)-activated derivative of horseradish peroxidase (HisProbe™-HRP Conjugate; Thermo Fisher Scientific, Waltham, MA, USA) was used for detection. Plasma samples were diluted 200-fold and 400-fold in phosphate-buffered saline containing 1% bovine serum albumin and 0.1% K_2_-EDTA and pipetted into mAb-coated 96-well plates (Clear Flat-Bottom Immuno Nonsterile 96-Well Plates, Thermo Fisher Scientific). A microplate washer (Immuno Wash™ 1575 Microplate Washer; Bio-Rad Laboratories, Hercules, CA, USA) was used for the washing process. Subsequently, o-Phenylenediamine (FUJIFILM Wako Pure Chemical Corporation, Osaka, Japan) and 30% H_2_O_2_ were used for the chromogenic reaction; the reaction was stopped with 3 M H_2_SO_4_. Plasma HRG levels were measured using a 96-well plate reader (Nivo™ 5S Multimode Plate Reader; PerkinElmer, Waltham, MA, USA) at an absorbance of 492 nm. A standard curve was established using serial dilutions of known amounts of purified HRG (prepared in-house). Each plasma sample was measured in duplicate, and plasma HRG levels were determined by averaging two independent assays. The intra and inter-assay coefficients of variability were 7.4% and 13%, respectively. WBC, CRP, PCT, and P-SEP levels were measured from the same blood used for the HRG-level measurements. PCT and P-SEP levels were determined using a chemiluminescent enzyme immunoassay (SRL, Tokyo, Japan). WBC and CRP levels were measured at the Clinical Chemistry Laboratory of Okayama University Hospital.

### Outcomes

The primary outcome was the HRG levels on POD 1 in the patients with and without postoperative complications. The secondary outcomes were the WBC, CRP, PCT, and P-SEP levels on POD 1 in the patients with and without postoperative complications, the association of HRG, WBC, CRP, PCT, and P-SEP with postoperative complications, and their ability to predict postoperative complications.

### Statistical analysis

The statistical approach was designed a priori. Multivariate, receiver operating characteristic (ROC) curve, and subgroup analyses were designed as post-hoc analyses. Categorical variables are expressed as numbers (percentiles) and compared using Fisher’s exact test. Continuous variables are expressed as median and interquartile ranges (IQRs, 25–75th percentiles) and compared using the Mann–Whitney U test or Kruskal–Wallis test. Furthermore, the Steel–Dwass test was used to compare the medians of continuous variables for the post-hoc analysis among the three groups. The differences in the means of continuous variables are expressed as differences in means and 95% confidence intervals (CIs) and were compared using t-tests. Cox proportional hazards models and ROC curve analysis were used to assess the ability of each biomarker to predict postoperative complications. The results of the Cox proportional hazards models are expressed as hazard ratio (HR), 95% CI, and Harrel C-index score. In the multivariate analysis, we adjusted for the presence of preoperative cardiovascular comorbidities, age, American Society of Anesthesiologists Physical Status Classification (ASA-PS), operative time, and the volume of intraoperative bleeding, which have been reported to be associated with postoperative complications [1, 2, 26–28]. To assess the association between HRG levels and postoperative complications, we utilized the Kaplan–Meier method and log-rank test by classifying patients into two groups using the cut-off levels obtained from the logistic regression ROC curve analysis. A two–sided *P*-value < 0.05 was considered statistically significant. Data were analyzed using JMP Pro 14.0.0 (SAS Institute Inc., Cary, NC, USA) and STATA 16.1 and 17.0 (Stata Corp LLC, College Station, TX, USA).

## Results

### Patient characteristics

**Fig. 1:**
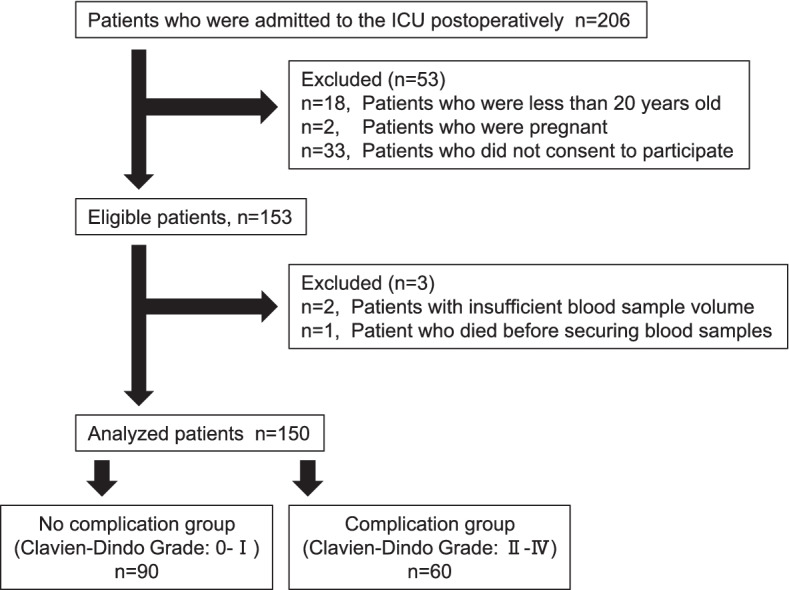
Patient flow chart. *ICU* intensive care unit

Patient characteristics are shown in Table 1. Eligible patients were prospectively included from September 17, 2020 to November 11, 2020. Figure 1 shows the patient flow. The data of 150 patients were included in the final sample and analyzed. None of the patients dropped out during the follow-up period. The patients were hospitalized in the departments of respiratory surgery (31 patients), neurosurgery (30 patients), hepato–biliary–pancreatic surgery (25 patients), gastrointestinal surgery (19 patients: esophageal surgery, 17 patients and colorectal surgery, two patients), cardiovascular surgery (12 patients), urology (nine patients), oral surgery (eight patients), otolaryngology (seven patients), orthopedic surgery (five patients), and breast–thyroid surgery (four patients).

**Table 1:** Patient characteristics

	No-complication group (*n* = 90)	Complication group (*n* = 60)	*P*-value
Preoperative factors			
Age (years), and median (IQR)	63.0 (51.0–72.0)	71.0 (60.3–76.0)	0.009
Male sex, n (%)	42 (46.7)	36 (60.0)	0.134
Comorbidity, n (%)			
Hypertension	40 (44.4)	30 (50.0)	0.510
Diabetes	22 (24.4)	10 (16.7)	0.311
Cardiovascular disease	11 (12.2)	16 (26.7)	0.030
Asthma	5 (5.6)	1 (1.7)	0.403
COPD	4 (4.4)	5 (8.3)	0.485
Liver cirrhosis	1 (1.1)	3 (5.0)	0.302
Chronic kidney disease (eGFR ≤ 50)	9 (10.0)	12 (20.0)	0.097
Acute infection	1 (1.1)	1 (1.7)	0.999
Autoimmune disease	7 (7.8)	4 (6.7)	0.999
Preoperative use of steroids	6 (6.7)	2 (3.3)	0.477
Preoperative use of heparin	1(1.1)	1(1.7)	0.999
ASA-PS ≥ III	17 (19.0)	23 (38.0)	0.014
Preoperative SOFA score, median (IQR)	0 (0–0)	0 (0–1)	0.085
Intraoperative factors			
Operative time, min, median (IQR)	249.5 (159.5–379.5)	398.5 (291.3–565.0)	< 0.001
Volume of bleeding (mL), median (IQR)	97.5 (5.0–267.5)	300 (120.0–888.8)	< 0.001
Fluid balance (mL), median (IQR)	1432.5 (962.5–2109.5)	2710.5 (1665.0–3920.8)	< 0.001
SAS, median (IQR)	7.0 (6.0–8.0)	6.0 (4.3–7.0)	< 0.001
Postoperative factors			
APACHE II score on admission to the ICU, median (IQR)	9 (7–11)	11 (9–14)	0.003
Postoperative SOFA score, median (IQR)	1.0 (0–3.0)	4.0 (2.3–5.8)	< 0.001
ICU stay (days), median (IQR)	2 (2–2)	4 (2–6)	< 0.001
Hospital stay (days), median (IQR)	16.0 (12.8–21.3)	29.0 (20.0–37.0)	< 0.001
Death within 28-days after surgery, n (%)	0	0	−

*APACHE II Score* Acute Physiology and Chronic Health Evaluation II Score, *ASA-PS* American Society Anesthesiologists Physical Status Classification, *COPD* chronic obstructive pulmonary disease, *GFR* estimated glomerular filtration rate, *ICU* intensive care unit, *IQR* interquartile range, *n* numbers, *SAS* surgical Apgar score, *SOFA Score* Sequential Organ Failure Assessment Score

Ninety patients with Clavien–Dindo grades 0–I were included in the ‘no-complication group’, and 60 patients with Clavien–Dindo grades II–IV were included in the ‘complication group’. The overall incidence of postoperative complications was 40%. In the complication group, 33 patients had Clavien–Dindo grade II, nine had grade III (eight patients, grade IIIa and one patient, grade IIIb), and 18 patients had grade IV (14 patients had grade IVa and four patients had grade IVb). Postoperative complications included hypotension in 11 patients, hemorrhage in seven patients, atelectasis/sputum excretion difficulty in six patients, thrombosis/embolism in six patients, intraabdominal abscess in six patients, and others in 24 patients. Twenty-seven patients underwent drainage or change in antibiotics due to infection. Details of postoperative complications are provided in Supplementary Table 1 in Additional file 1. The distribution of the participants in the no-complication and complication groups per clinical department is shown in Supplementary Table 2 in Additional file 2. Postoperative complications developed on median POD 3 (IQR, 1–5 days).

Regarding preoperative factors, patients in the complication group had significantly higher age, higher incidence of cardiovascular diseases, and more instances of ASA-PS ≥ III than those in the no-complication group. Regarding the intraoperative factors, patients in the complication group had a significantly longer operative time and greater amount of bleeding and intraoperative fluid balance than those in the no-complication group. The SAS in the complication group was significantly lower than that in the no-complication group. Regarding postoperative factors, ICU and hospital stays in the complication group were significantly longer than those in the no-complication group. Among all patients, no deaths occurred within the first 28 days after surgery.

### HRG and other biomarker levels

**Fig. 2:**
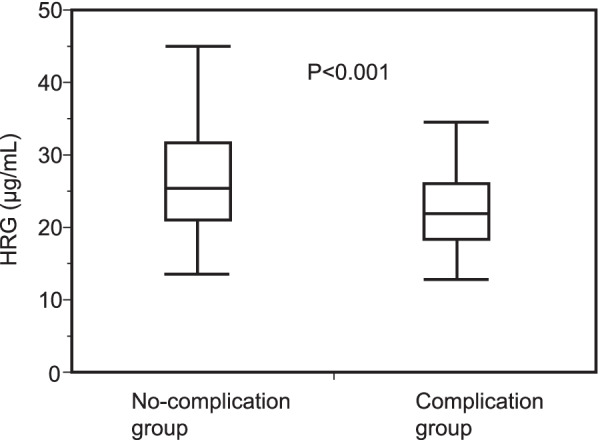
Plasma HRG levels on postoperative day 1 in the groups with and without postoperative complications. The box shows the median, 25th, and 75th percentiles. Bars represent the 5th and 95th percentiles. The Mann–Whitney U test was used. *P* < 0.05 was considered significant. *HRG* histidine-rich glycoprotein, *POD 1* postoperative day 1

Figure 2 shows that the HRG levels on POD 1 in the complication group (21.50 µg/mL [IQR, 18.12–25.74 µg/mL]) were significantly lower than those in the no-complication group (25.46 µg/mL [IQR, 21.05–31.63 µg/mL]) (*P* < 0.001). Table 2 shows the WBC, CRP, PCT, and P-SEP levels on POD 1. The CRP, PCT, and P-SEP levels on POD 1 in the complication group were higher than those in the no-complication group. The WBC levels on POD 1 were not significantly different between the two groups.

**Table 2:** White blood cell, C-reactive protein, procalcitonin, and presepsin levels on postoperative day 1

	No-complication group (*n* = 90)	Complication group (*n* = 60)	*P*-value
WBC (/µL)	9265.0 (7275.0–11,927.5)	9585.0 (7557.5–11,267.5)	0.877
CRP (mg/dL)	3.32 (1.66–5.78)	4.68 (2.75–6.49)	0.036
PCT (ng/mL)	0.06 (0.03–0.18)	0.25 (0.12–0.75)	< 0.001
P-SEP (pg/mL)	447.5 (355.3–597.5)	778.5 (578.8–1047.5)	< 0.001

Expressed as median (IQR)

*CRP* C-reactive protein, *IQR* interquartile range, *P-SEP* presepsin, *PCT* procalcitonin, *WBC* white blood cell

### Biomarkers’ ability to predict postoperative complications

Table 3 shows the association between biomarkers and postoperative complications. In the univariate analyses, we found that the HRG, CRP, PCT, and P-SEP levels were significantly associated with postoperative complications, but the WBC levels were not. Furthermore, the Harrell C-index scores for postoperative complications were HRG, 0.65; WBC, 0.50; CRP, 0.59; PCT, 0.73; and P-SEP, 0.73. In multivariate analyses, after adjustment for confounding factors, such as age, presence of preoperative cardiovascular comorbidities, ASA-PS, operative time, and volume of intraoperative bleeding, only HRG and P-SEP were found to be independent predictors of complications.

**Table 3:** Associations between biomarkers and postoperative complications

Variables	Univariate analysis			Multivariate analysis	
	Unadjusted HR (95% CI)	*P*-value	Harrell C-index	Adjusted HR (95% CI)	*P*-value
HRG	0.92 (0.88–0.96)	< 0.001	0.65	0.94 (0.90–0.99)	0.014
WBC/1000	0.98 (0.90–1.06)	0.554	0.50	0.97 (0.89–1.06)	0.484
CRP	1.08 (1.003–1.16)	0.042	0.59	1.03 (0.96–1.11)	0.453
PCT	1.06 (1.01–1.11)	0.019	0.73	1.03 (0.98–1.09)	0.284
P-SEP/100	1.18 (1.12–1.24)	< 0.001	0.73	1.13 (1.06–1.20)	< 0.001

*HR* hazard ratio, *Adjusted HR* hazard ratio adjusted for age, presence of preoperative cardiovascular comorbidities, American Society Anesthesiologists Physical Status Classification, operative time, and volume of intraoperative bleeding, *CRP* C-reactive protein, *HRG* histidine-rich glycoprotein, *P-SEP* presepsin, *PCT* procalcitonin, *WBC* white blood cell

**Fig. 3:**
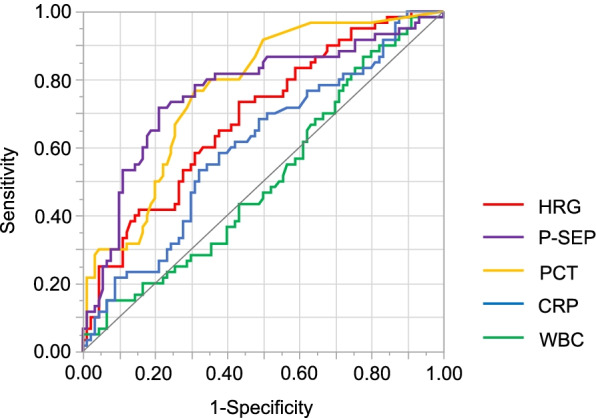
Receiver operating characteristic curves for predicting postoperative complications. Receiver operating characteristic curves of HRG, P-SEP, PCT, CRP, and WBC. *CRP* C-reactive protein, *HRG* histidine-rich glycoprotein, *P-SEP* presepsin, *PCT* procalcitonin, *WBC* white blood cell

**Fig. 4:**
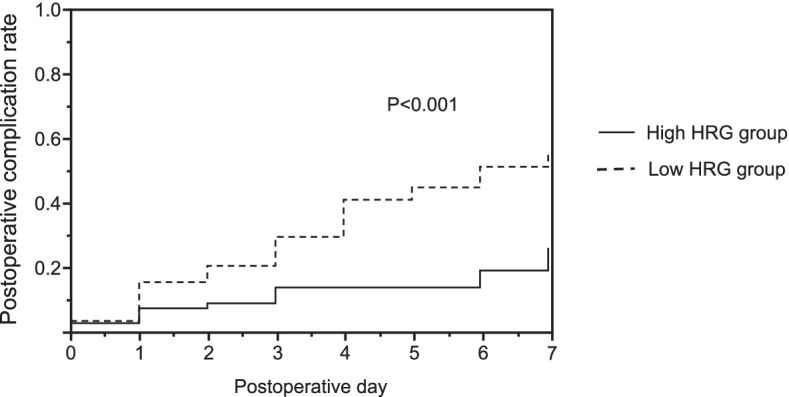
Kaplan–Meier curves. Patients were classified into two groups of high (*n* = 67) and low (*n* = 83) HRG levels, using a cut-off level of 24.21 µg/mL. The Kaplan–Meier method and log-rank test were used. *P* < 0.05 was considered significant. *HRG* histidine-rich glycoprotein

Furthermore, we performed ROC curve analysis to compare the predictive ability of each biomarker. The area under curve (AUC) was HRG, 0.69; P-SEP, 0.76; PCT, 0.77; CRP, 0.60; and WBC, 0.51. The AUC for HRG was significantly higher than that of WBC (*P* = 0.005). There was no significant difference among the AUCs of HRG, P-SEP, PCT, and CRP. The sensitivity and specificity of the HRG levels to predict postoperative complications at the cut-off level of 24.21 µg/mL were 0.73 and 0.57, respectively (Fig. 3). Furthermore, when the analyzed patients were divided into a high-HRG group and a low-HRG group using this cut-off level, the postoperative complication rate of the low HRG group (*n* = 83) was significantly higher than that of the high HRG group (*n* = 67) (Fig. 4).

### HRG levels between the subgroups with and without postoperative complications by clinical department

We examined the differences in the means of the HRG levels on POD 1 between the no-complication and complication groups in patients of the respiratory surgery (*n* = 31) and hepato–biliary–pancreatic surgery (*n* = 25) departments. The difference was set as [means of HRG levels on POD 1 in the no-complication group] minus [means of HRG levels on POD 1 in the complication group] and was found to be nonsignificant. For details, see Supplementary Fig. 1 in Additional file 3. The number of patients in the other clinical departments did not suffice for examining between-group differences.

### HRG and other biomarker levels in the groups with and without postoperative infectious complications

We classified the complication group into two subgroups: those who developed infectious complications (infectious-complication group, *n* = 27) and those who developed non-infectious complications (non-infectious-complication group, *n* = 33). HRG and other biomarker levels on POD 1 were compared between the no-complication, non-infectious-complication, and infectious-complication groups. The HRG, PCT, and P-SEP levels on POD 1 were significantly different among the three groups (HRG, *P* < 0.001; PCT, *P* < 0.001; P-SEP, *P* < 0.001, respectively). However, there was no significant difference in HRG, PCT, and P-SEP levels on POD 1 between the infectious- and non-infectious-complication groups. The CRP and WBC levels on POD 1 were not significantly different among the three groups. For details, see Supplementary Fig. 2 in Additional file 4.

### HRG and other biomarker levels and severity of postoperative complications

We divided the complication group into two subgroups: those classified as Clavien–Dindo grade II (mild-complication group; *n* = 33) and those classified as Clavien–Dindo grades III and IV (severe-complication group; *n* = 27). The HRG and other biomarker levels on POD 1 were compared between the no-complication, mild-complication, and severe-complication groups. The HRG, PCT, and P-SEP levels on POD 1 were significantly different among the three groups (HRG, *P* < 0.001; PCT, *P* < 0.001; P-SEP, *P* < 0.001, respectively). Furthermore, the P-SEP levels on POD 1 in the severe-complication group were higher than those in the mild-complication group. However, there was no significant difference in the HRG and PCT levels on POD 1 between the mild- and severe-complication groups. The CRP and WBC levels on POD 1 were not significantly different among the three groups. For details, see Supplementary Fig. 3 in Additional file 5.

## Discussion

In this study, we found that the HRG levels on POD 1 were significantly lower in the complication group than in the no-complication group. Furthermore, the ability of HRG to predict postoperative complications was superior to that of WBC and CRP, and similar to that of PCT and P-SEP. However, the difference in HRG levels on POD 1 between the no-complication and complication groups was not significant for the patients of the respiratory and hepato–biliary–pancreatic surgery departments, and there were no significant differences in HRG levels on POD 1 between the infectious- and non-infectious-complication groups and mild- and severe-complication groups.

Previous studies have shown that HRG levels negatively correlate with CRP levels in patients with acute inflammation; therefore, HRG may function as a negative acute phase reactant [16, 29]. Previous clinical studies have reported that a decrease in HRG levels may be a biomarker for sepsis [16, 20], ventilator-associated pneumonia [21], preeclampsia [22], and coronavirus disease 2019 [23]. We found that a decrease in HRG levels on POD 1 might predict postoperative complications, and the ability of HRG to predict postoperative complications was more strongly associated with postoperative complications than that of WBC and CRP and had an association strength similar to that of PCT and P-SEP. Furthermore, the HRG levels could independently predict postoperative complications in the multivariate analyses. Thus, our study showed that the HRG level may be a novel and independent biomarker for predicting postoperative complications.

However, there were no significant differences in HRG levels on POD 1 between patients who developed and did not develop postoperative complications in the respiratory and hepato–biliary–pancreatic surgery departments. It could be that decrease in HRG levels on POD 1 might not predict postoperative complications in some clinical departments; however, the detection power may have been insufficient because of the low number of samples. Further studies could confirm this hypothesis. Furthermore, there were no significant differences in HRG levels on POD 1 between patients who developed infectious complications and those who developed non-infectious complications and between the mild- and severe-complication groups. In contrast, in our previous study with patients who had systemic inflammatory response syndrome (SIRS), we concluded that HRG may be a biomarker for detecting infection and may be useful for evaluating severity [16]. This contradictory result may be attributable to differences in the studied populations, i.e., patients who had undergone surgery and patients with SIRS.

The main strength of this study is that it showed the association between HRG levels and postoperative complications. We believe that patients with very low postoperative HRG levels are at high risk of developing postoperative complications, and clinicians need to follow them more closely.

This study had several limitations. First, it was a single-center study. Therefore, it is unclear whether our findings can be applied to other populations. Second, many of the included patients had cancer. It has been reported that HRG may prevent the development of tumors [30], and the levels vary across breast, ovarian, and lung cancers [31–33]. The variations in HRG levels in other cancers are unknown. These patient characteristics may have influenced the results. Third, the time from the end of surgery to specimen collection varied across patients because we used residual blood samples collected for routine blood tests in the morning of POD 1. This difference in time may have influenced the postoperative changes in HRG levels. Fourth, HRG levels were measured only on POD 1. HRG levels before surgery and the time course of HRG levels after surgery are unknown. Further studies are needed to examine these issues. Fifth, the postoperative complications in this study include surgical complications, which are unrelated to the intrinsic physiology of the patients. Further study limited to medical complications related to the bioactivity of HRG is needed. Sixth, the study included postoperative patients from a variety of clinical departments, with differences in the originating departments between the two groups. This background may have influenced the results. Further studies limited to specific departments are needed. Seventh, the differences in HRG levels between patients with and without postoperative complications were smaller than expected. We expected a difference of approximately 20 µg/mL between the two groups, in reference to the results of our previous study [16]. However, accurate prediction of differences in HRG levels on POD 1 between the two groups was challenging because of the paucity of studies in which postoperative HRG levels have been measured. Hence, our power calculation may have been ineffective.

## Conclusions

The HRG levels on POD 1 in patients who developed postoperative complications were significantly lower than those in patients who did not develop postoperative complications. Furthermore, the ability of HRG to predict postoperative complications was superior to that of WBC and CRP and similar to that of PCT and P-SEP. Thus, HRG levels may be useful biomarkers for predicting postoperative complications. Future studies are needed on the usefulness of HRG in predicting postoperative complications based on clinical departments and complications.

## Supplementary Information

**Additional file 1: Supplementary Table 1.** Postoperative complications (extended Clavien–Dindoclassification grade ≥ II). Details of postoperative complications.

**Additional file 2: Supplementary Table 2.** Distribution of the patients in the no-complication and complication groups according to clinical department of surgery. Detailed description of the distribution of the patients in the no-complication group and complication group according to the clinical department of surgery.

**Additional file 3: Supplementary Fig. 1.** Differences in the mean of HRG levels on postoperative day 1 in the groups with and without postoperative complications by department of surgery. Differences in the mean of HRG levels on postoperative day 1 in the groups with and without postoperative complications have been illustrated according to the clinical department of surgery.

**Additional file 4: Supplementary Fig. 2.** Levels of plasma biomarkers in the groups with and without postoperative infectious complications. Illustration showing the comparison of the levels of plasma biomarkers in the groups with and without postoperative infectious complications.

**Additional file 5: Supplementary Fig. 3.** Levels of plasma biomarkers and severity of postoperative complications. Illustration of the comparison of the levels of plasma biomarkers and severity of postoperative complications among the groups.

## Data Availability

The datasets generated and analyzed during the present study are available from the corresponding author on reasonable request.

## Abbreviations

ASA-PS: American Society of Anesthesiologists Physical Status Classification
AUC: Area under the curve
CI: Confidence interval
CRP: C-reactive protein
HR: Hazard ratio
HRG: Histidine-rich glycoprotein
ICU: Intensive care unit
IQR: Interquartile range
mAb: Monoclonal antibody
PCT: Procalcitonin
POD: Postoperative day
P-SEP: Presepsin
ROC: Receiver operating characteristic
SAS: Surgical Apgar score
SIRS: Systemic inflammatory response syndrome
WBC: White blood cell
