# Supplementary material for: Histidine-rich glycoprotein as a novel predictive biomarker of postoperative complications in intensive care unit patients: a prospective observational study
Source: BMC Anesthesiol. 2022 Jul 20;22:232. doi: 10.1186/s12871-022-01774-7 (PMC9296898; doi:10.1186/s12871-022-01774-7)
Supplement: Supplementary file 1 — Additional file 1: Supplementary Table 1. Postoperative complications (extended Clavien–Dindoclassification grade ≥ II). Details of postoperative complications. [file 12871_2022_1774_MOESM1_ESM.pdf]

Supplementary Table 1. Postoperative complications (extended Clavien–Dindo classification grade ≥ II)

Postoperative complication	*n*
Haemorrhage	7
Atelectasis/sputum excretion difficulty	6
Thrombosis/embolism	6
Intraabdominal abscess	6
Supraventricular arrhythmia	4
Ventricular arrhythmia	2
Pleural effusion	3
Pancreatic fistula	3
Pneumonia	2
Chylothorax	1
Ascites	2
Delayed gastric emptying	1
Biliary fistula	1
Gastrointestinal anastomotic leak	1
Wound infection	1
Others	
Hypotension	11
Urinary tract infection	2
Infection (focus unknown)	1

n numbers
