# Supplementary material for: Histidine-rich glycoprotein as a novel predictive biomarker of postoperative complications in intensive care unit patients: a prospective observational study
Source: BMC Anesthesiol. 2022 Jul 20;22:232. doi: 10.1186/s12871-022-01774-7 (PMC9296898; doi:10.1186/s12871-022-01774-7)
Supplement: Supplementary file 2 — Additional file 2: Supplementary Table 2. Distribution of the patients in the no-complication and complication groups according to clinical department of surgery. Detailed description of the distribution of the patients in the no-complication group and complication group according to the clinical department of surgery. [file 12871_2022_1774_MOESM2_ESM.pdf]

Supplementary Table 2. Distribution of the patients in the no-complication and complication groups according to clinical department of surgery

Clinical department of surgery, *n*	No complication group (n=90)	Complication group (n=60)
Respiratory surgery (n=31)	25	6
Neurosurgery (n=30)	29	1
Hepato-biliary-pancreatic surgery (n=25)	10	15
Gastrointestinal surgery (n=19)	4	15
Cardiovascular surgery (n=12)	3	9
Urology (n=9)	3	6
Oral surgery (n=8)	5	3
Otolaryngology (n=7)	5	2
Orthopaedic surgery (n=5)	2	3
Breast-thyroid surgery (n=4)	4	0

n numbers
