# Supplementary material for: Histidine-rich glycoprotein as a novel predictive biomarker of postoperative complications in intensive care unit patients: a prospective observational study
Source: BMC Anesthesiol. 2022 Jul 20;22:232. doi: 10.1186/s12871-022-01774-7 (PMC9296898; doi:10.1186/s12871-022-01774-7)
Supplement: Supplementary file 3 — Additional file 3: Supplementary Fig. 1. Differences in the mean of HRG levels on postoperative day 1 in the groups with and without postoperative complications by department of surgery. Differences in the mean of HRG levels on postoperative day 1 in the groups with and without postoperative complications have been illustrated according to the clinical department of surgery. [file 12871_2022_1774_MOESM3_ESM.pdf]

Supplementary Fig.1 Differences in the mean of HRG levels on postoperative day 1 in the groups with and without postoperative complications by department of surgery

Department, n	Difference in mean HRG levels (μg/mL)
All, n=150	5.49 (2.71−8.27)
Respiratory surgery, n=31	4.57 (-4.45−13.60)
Hepato-biliary-pancreatic surgery, n=25	1.93 (-1.97−5.83)

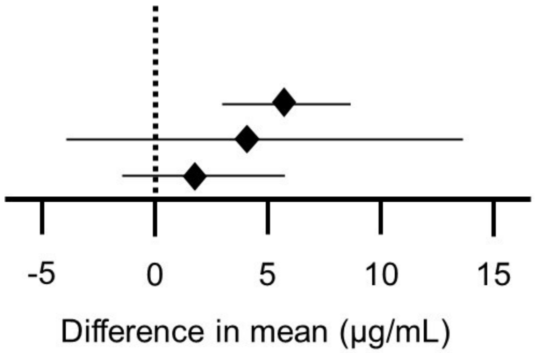

Difference: [HRG levels on POD 1 in the no-complication group] minus [HRG levels on POD 1 in the complication group]
Differences are presented as mean (diamond shape) and 95% CI (whiskers).
T-tests were used.
CI confidence interval, *HRG* histidine-rich glycoprotein, *n* number, *POD 1* postoperative day 1
