# Supplementary material for: Histidine-rich glycoprotein as a novel predictive biomarker of postoperative complications in intensive care unit patients: a prospective observational study
Source: BMC Anesthesiol. 2022 Jul 20;22:232. doi: 10.1186/s12871-022-01774-7 (PMC9296898; doi:10.1186/s12871-022-01774-7)
Supplement: Supplementary file 4 — Additional file 4: Supplementary Fig. 2. Levels of plasma biomarkers in the groups with and without postoperative infectious complications. Illustration showing the comparison of the levels of plasma biomarkers in the groups with and without postoperative infectious complications. [file 12871_2022_1774_MOESM4_ESM.pdf]

Supplementary Fig.2 Levels of plasma biomarkers in the groups with and without postoperative infectious complications

(a)

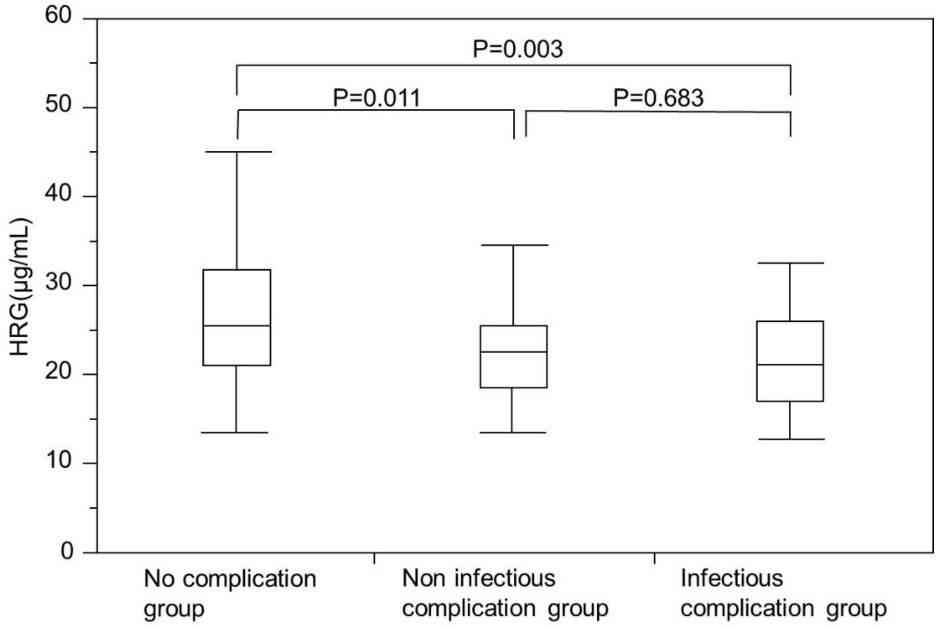

(b)

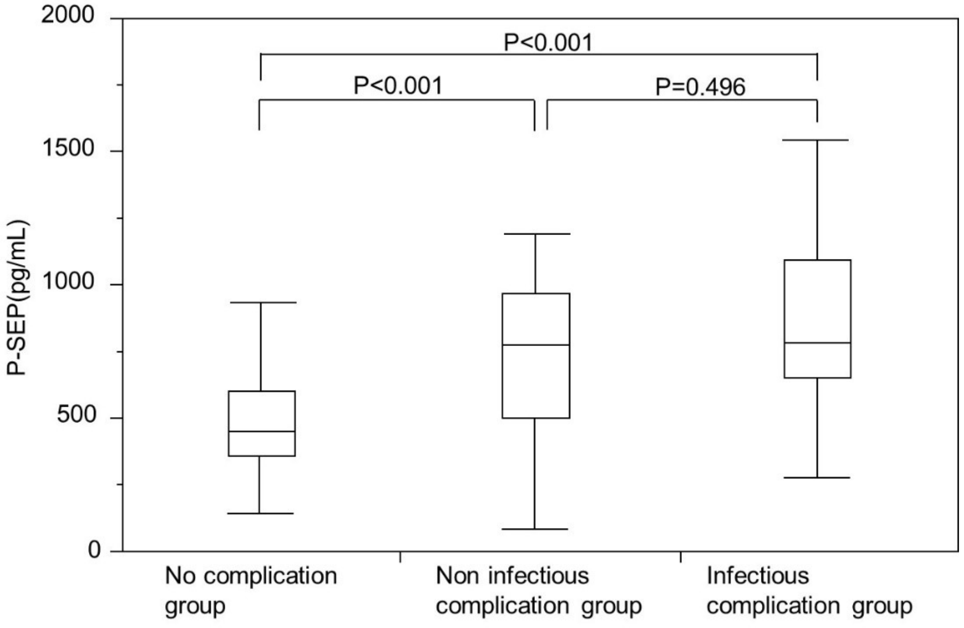

(c)

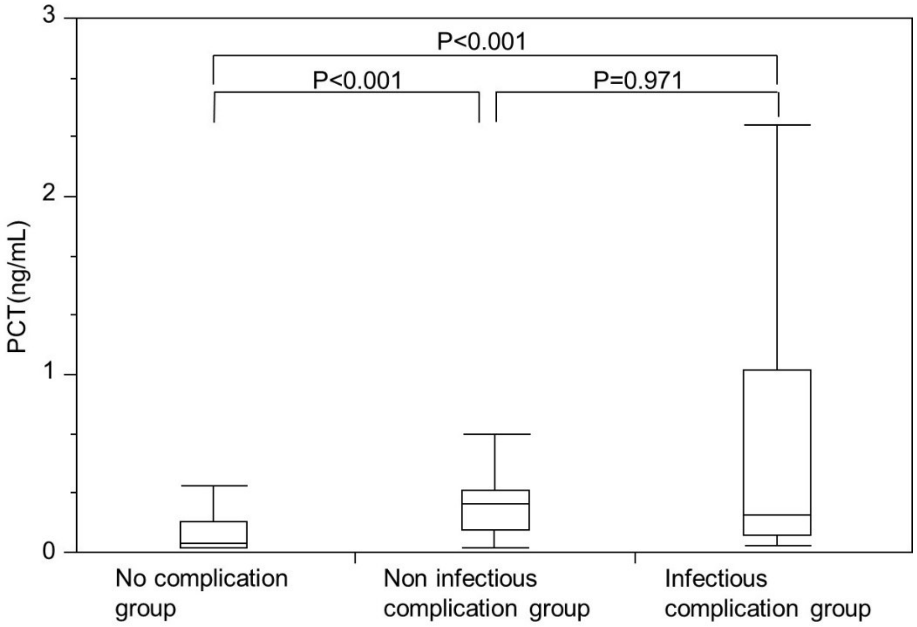
P<0.001
P<0.001
P=0.971
3
2
1
0
PCT(ng/mL)
No complication group
Non infectious complication group
Infectious complication group

(d)

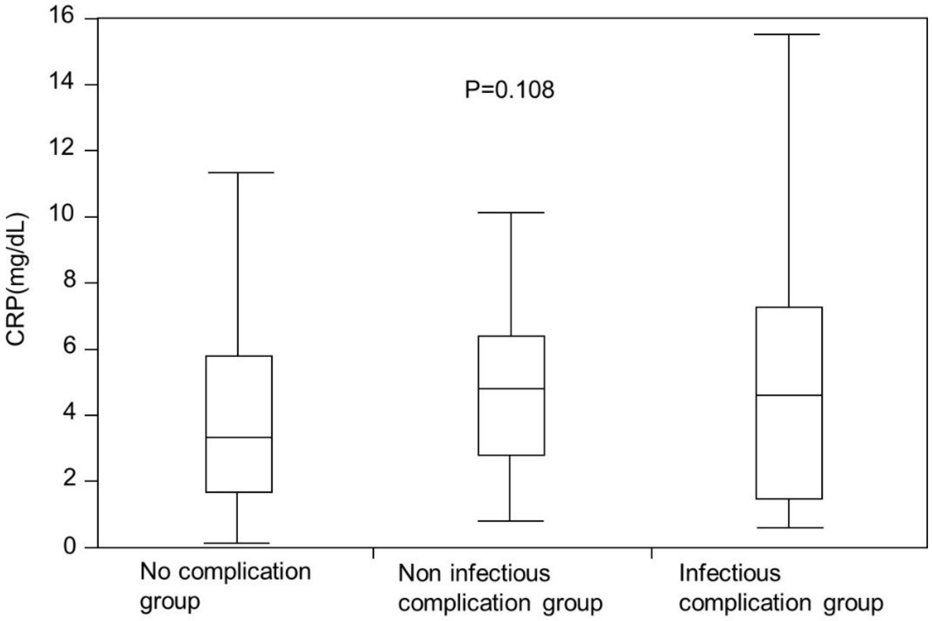
P=0.108
16
14
12
10
8
6
4
2
0
CRP(mg/dL)
No complication group
Non infectious complication group
Infectious complication group

(e)

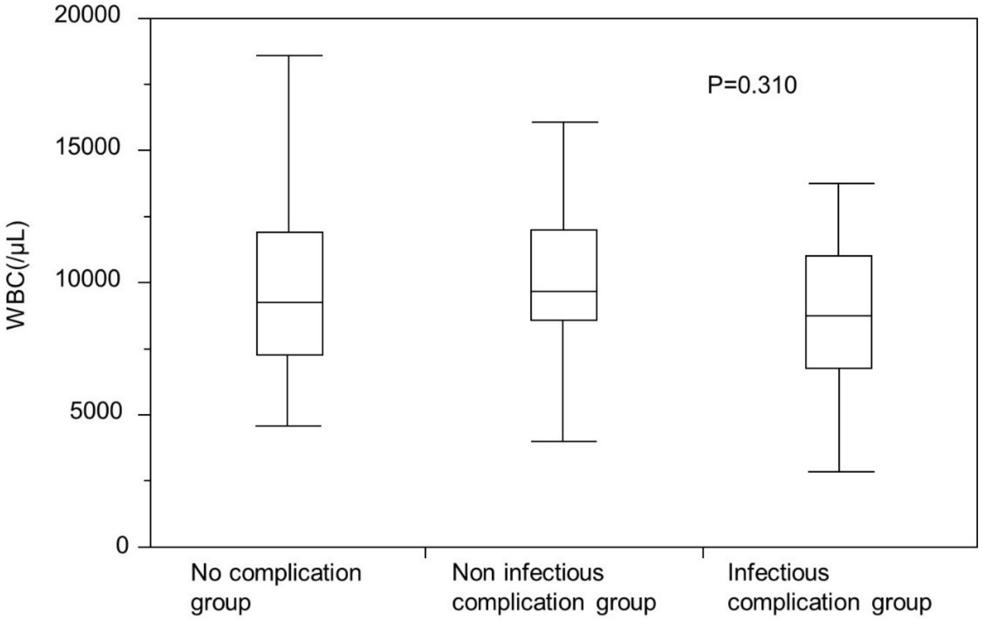

We compared each biomarker level among the no-complication group (n=90), non-infectious-complication group (n=33), and infectious-complication group (n=27).

(a) HRG; (b) P-SEP; (c) PCT; (d) CRP; (e) WBC levels among the three groups.

The box shows the median, 25th, and 75th percentiles. Bar represent the 5th and 95th percentiles.

The Kruskal–Wallis and Steel–Dwass tests were used.

P-value <0.05 was considered significant.

CRP C-reactive protein, *HRG* histidine-rich glycoprotein, *P-SEP* presepsin, *PCT* procalcitonin, *WBC* white blood cell
