# Supplementary material for: Histidine-rich glycoprotein as a novel predictive biomarker of postoperative complications in intensive care unit patients: a prospective observational study
Source: BMC Anesthesiol. 2022 Jul 20;22:232. doi: 10.1186/s12871-022-01774-7 (PMC9296898; doi:10.1186/s12871-022-01774-7)
Supplement: Supplementary file 5 — Additional file 5: Supplementary Fig. 3. Levels of plasma biomarkers and severity of postoperative complications. Illustration of the comparison of the levels of plasma biomarkers and severity of postoperative complications among the groups. [file 12871_2022_1774_MOESM5_ESM.pdf]

Supplementary Fig.3 Levels of plasma biomarkers and severity of postoperative complications

(a)

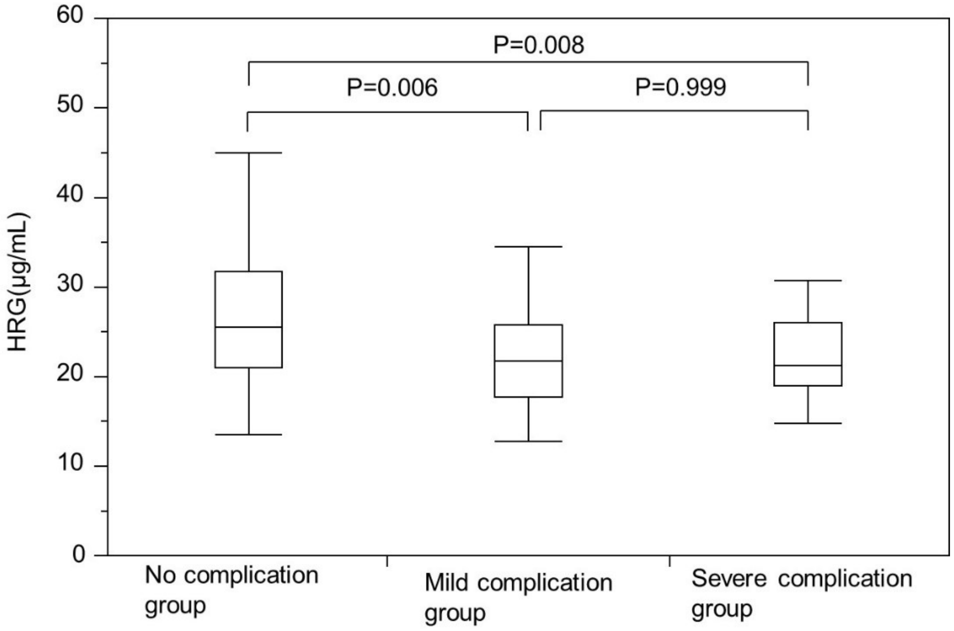

(b)

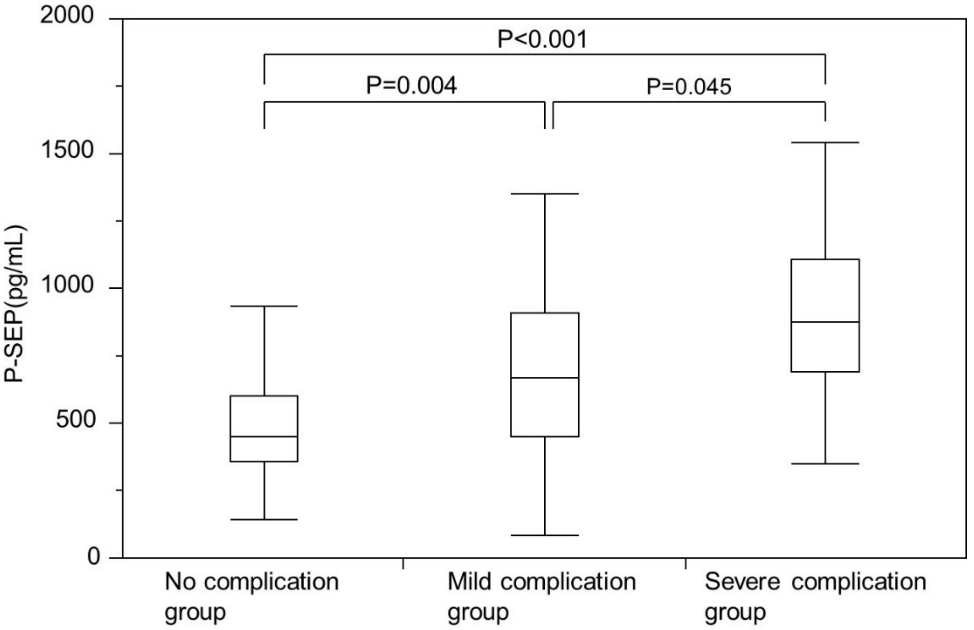

(c)

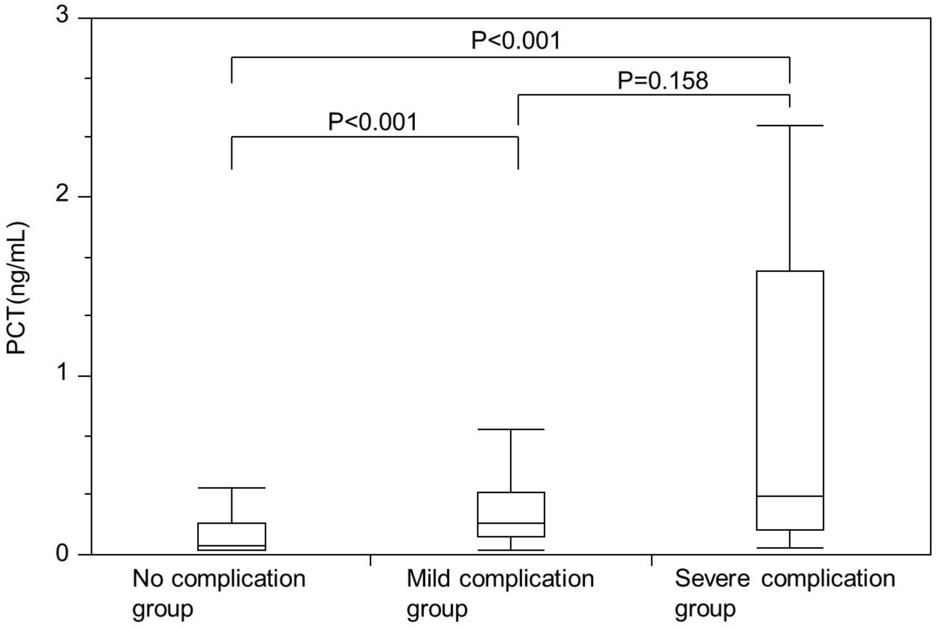
P<0.001
P=0.158
P<0.001
3
2
1
0
PCT(ng/mL)
No complication group
Mild complication group
Severe complication group

(d)

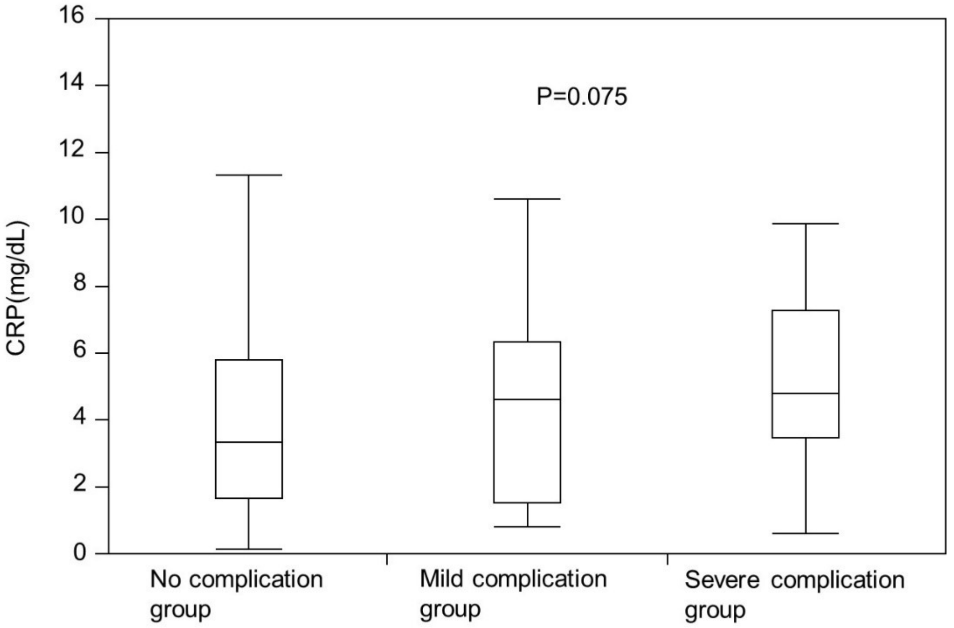
16
14
12
10
8
6
4
2
0
P=0.075
CRP(mg/dL)
No complication group
Mild complication group
Severe complication group

(e)

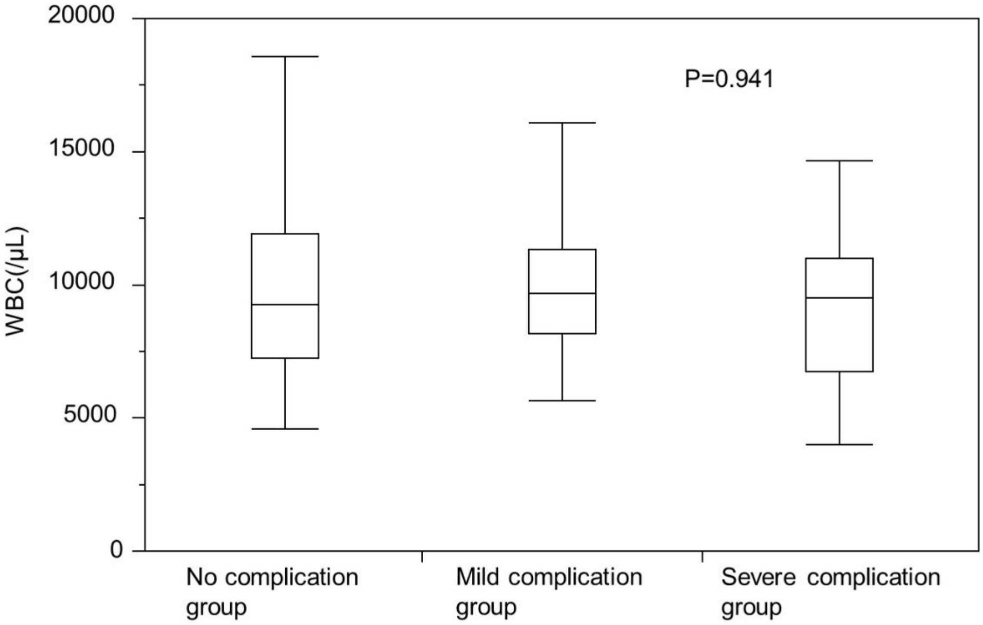

We compared each biomarker level among the no-complication group (n=90), mild-complication group (n=33), and severe-complication group (n=27).

(a) HRG; (b) P-SEP; (c) PCT; (d) CRP; (e) WBC levels among the three groups.

The box shows the median, 25th, and 75th percentiles. Bar represent the 5th and 95th percentiles.

The Kruskal–Wallis and Steel–Dwass tests were used.

P-value <0.05 was considered significant.

CRP C-reactive protein, *HRG* histidine-rich glycoprotein, *P-SEP* presepsin, *PCT* procalcitonin, *WBC* white blood cell
